# Supplementary material for: Electrically driven spin torque and dynamical Dzyaloshinskii-Moriya interaction in magnetic bilayer systems
Source: Sci Rep. 2019 Jul 2;9:9528. doi: 10.1038/s41598-019-46009-x (PMC6606575; doi:10.1038/s41598-019-46009-x)
Supplement: Supplementary file 1 — Supplementary Material [file 41598_2019_46009_MOESM1_ESM.pdf]

Supplementary Material for “Electrically driven spin torque and dynamical Dzyaloshinskii-Moriya interaction in magnetic bilayer systems”

Akihito Takeuchi[1,*], **Shigeyasu Mizushima**[2], **and Masahito Mochizuki**[1,2,3,*]

[1]Department of Physics and Mathematics, Aoyama Gakuin University, Sagamihara, Kanagawa 252-5258, Japan
[2]Department of Applied Physics, Waseda University, Okubo, Shinjuku-ku, Tokyo 169-8555, Japan
[3]PRESTO, Japan Science and Technology Agency, Kawaguchi, Saitama 332-0012, Japan
*akihito@phys.aoyama.ac.jp (A.T.); masa_mochizuki@waseda.jp (M.M.)

Calculation of Ψ-electron spin density $S^{\pm}$

We provide details of the derivation of the Ψ-electron spin density $S^{\pm}$. In Fig. S1, we present the Feynman diagrams associated with the Ψ-electron spin density induced by the time-dependent RSOI. Here the vertex corrections due to the nonmagnetic impurity scatterings are not considered because they are negligible in the present case.

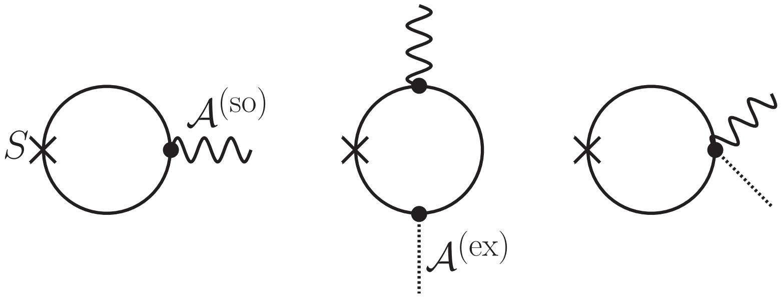

Figure S1. Diagrammatic representations of the dominant contributions of the Ψ-electron spin density S. The solid, wavy, and dotted lines represent the Green function, the RSOI $\mathscr{A}^{(\mathrm{so})}$, and the exchange interaction $\mathscr{A}^{(\mathrm{ex})}$ in the rotated spin frame, respectively.

Expanding the lesser component with respect to $\boldsymbol{q}$ and Ω, the spin density $S^{\pm}(\boldsymbol{q},\Omega)$ is written

$$
\begin{aligned}
S^{\pm}(\boldsymbol{q},\Omega) \;=\;& \frac{ie\hbar^4}{m_{\mathrm{e}}^2}\Big\{ \pm q_\nu \mathscr{A}_\mu^{(\mathrm{so})\pm}(\boldsymbol{q},\Omega) - \frac{2e}{\hbar}\sum_{\boldsymbol{q}'}\Big[\mathscr{A}_\mu^{(\mathrm{so})\pm}(\boldsymbol{q}',\Omega)\mathscr{A}_\nu^{(\mathrm{ex})z}(\boldsymbol{q}-\boldsymbol{q}') + \mathscr{A}_\mu^{(\mathrm{so})z}(\boldsymbol{q}',\Omega)\mathscr{A}_\nu^{(\mathrm{ex})\pm}(\boldsymbol{q}-\boldsymbol{q}')\Big]\Big\} \\
& \times \sum_{\sigma=\pm}\sigma\sum_{\boldsymbol{k}}\sum_{\omega} f_\omega k_\mu k_\nu \Big[g^{\mathrm{a}}_{\boldsymbol{k},\omega,-\sigma}(g^{\mathrm{a}}_{\boldsymbol{k},\omega,\sigma})^2 - g^{\mathrm{r}}_{\boldsymbol{k},\omega,-\sigma}(g^{\mathrm{r}}_{\boldsymbol{k},\omega,\sigma})^2\Big] \\
& + \frac{ie\hbar^4}{2m_{\mathrm{e}}^2}\Omega\Big[\pm q_\nu \mathscr{A}_\mu^{(\mathrm{so})\pm}(\boldsymbol{q},\Omega) - \frac{2e}{\hbar}\sum_{\boldsymbol{q}'}\mathscr{A}_\mu^{(\mathrm{so})\pm}(\boldsymbol{q}',\Omega)\mathscr{A}_\nu^{(\mathrm{ex})z}(\boldsymbol{q}-\boldsymbol{q}')\Big] \\
& \times \sum_{\sigma=\pm}(\sigma\mp 1)\sum_{\boldsymbol{k}}\sum_{\omega} k_\mu k_\nu \Big\{\frac{df_\omega}{d\omega}\Big[g^{\mathrm{r}}_{\boldsymbol{k},\omega,\sigma}(g^{\mathrm{a}}_{\boldsymbol{k},\omega,-\sigma})^2 - (g^{\mathrm{r}}_{\boldsymbol{k},\omega,\sigma})^2 g^{\mathrm{a}}_{\boldsymbol{k},\omega,-\sigma} \\
& + \frac{1}{2}g^{\mathrm{a}}_{\boldsymbol{k},\omega,\sigma}(g^{\mathrm{a}}_{\boldsymbol{k},\omega,\sigma} - g^{\mathrm{a}}_{\boldsymbol{k},\omega,-\sigma})g^{\mathrm{a}}_{\boldsymbol{k},\omega,-\sigma} + \frac{1}{2}g^{\mathrm{r}}_{\boldsymbol{k},\omega,\sigma}(g^{\mathrm{r}}_{\boldsymbol{k},\omega,\sigma} - g^{\mathrm{r}}_{\boldsymbol{k},\omega,-\sigma})g^{\mathrm{r}}_{\boldsymbol{k},\omega,-\sigma}\Big] \\
& - \hbar f_\omega\Big[g^{\mathrm{a}}_{\boldsymbol{k},\omega,-\sigma}g^{\mathrm{a}}_{\boldsymbol{k},\omega,\sigma}(g^{\mathrm{a}}_{\boldsymbol{k},\omega,\sigma} - g^{\mathrm{a}}_{\boldsymbol{k},\omega,-\sigma})^2 + (g^{\mathrm{a}}_{\boldsymbol{k},\omega,-\sigma})^2(g^{\mathrm{a}}_{\boldsymbol{k},\omega,\sigma})^2 \\
& - g^{\mathrm{r}}_{\boldsymbol{k},\omega,-\sigma}g^{\mathrm{r}}_{\boldsymbol{k},\omega,\sigma}(g^{\mathrm{r}}_{\boldsymbol{k},\omega,\sigma} - g^{\mathrm{r}}_{\boldsymbol{k},\omega,-\sigma})^2 - (g^{\mathrm{r}}_{\boldsymbol{k},\omega,-\sigma})^2(g^{\mathrm{r}}_{\boldsymbol{k},\omega,\sigma})^2\Big]\Big\} \\
& + \frac{ie^2\hbar^3}{m_{\mathrm{e}}^2}\Omega\sum_{\boldsymbol{q}'}\mathscr{A}_\mu^{(\mathrm{so})z}(\boldsymbol{q}',\Omega)\mathscr{A}_\nu^{(\mathrm{ex})\pm}(\boldsymbol{q}-\boldsymbol{q}') \\
& \times \sum_{\sigma=\pm}(\sigma\mp 1)\sum_{\boldsymbol{k}}\sum_{\omega} k_\mu k_\nu \Big\{\frac{df_\omega}{d\omega}\Big[g^{\mathrm{r}}_{\boldsymbol{k},\omega,\sigma}g^{\mathrm{a}}_{\boldsymbol{k},\omega,-\sigma}g^{\mathrm{a}}_{\boldsymbol{k},\omega,\sigma} - g^{\mathrm{r}}_{\boldsymbol{k},\omega,-\sigma}g^{\mathrm{r}}_{\boldsymbol{k},\omega,\sigma}g^{\mathrm{a}}_{\boldsymbol{k},\omega,-\sigma}
\end{aligned}
$$

$$-\frac{1}{2}g^{\rm a}_{\boldsymbol{k},\omega,\sigma}(g^{\rm a}_{\boldsymbol{k},\omega,\sigma}-g^{\rm a}_{\boldsymbol{k},\omega,-\sigma})g^{\rm a}_{\boldsymbol{k},\omega,-\sigma}-\frac{1}{2}g^{\rm r}_{\boldsymbol{k},\omega,\sigma}(g^{\rm r}_{\boldsymbol{k},\omega,\sigma}-g^{\rm r}_{\boldsymbol{k},\omega,-\sigma})g^{\rm r}_{\boldsymbol{k},\omega,-\sigma}\Bigg]$$
$$-\hbar f_\omega\Big[(g^{\rm a}_{\boldsymbol{k},\omega,-\sigma})^2(g^{\rm a}_{\boldsymbol{k},\omega,\sigma})^2-(g^{\rm r}_{\boldsymbol{k},\omega,-\sigma})^2(g^{\rm r}_{\boldsymbol{k},\omega,\sigma})^2\Big]\Bigg\}. \qquad \text{(S.1)}$$

Using the following relation,

$$\pm i\nabla_\nu \mathscr{A}^{({\rm so})\pm}_\mu+\frac{2e}{\hbar}\mathscr{A}^{({\rm so})\pm}_\mu\mathscr{A}^{({\rm ex})z}_\nu=\frac{2e}{\hbar}\mathscr{A}^{({\rm so})z}_\mu\mathscr{A}^{({\rm ex})\pm}_\nu, \qquad \text{(S.2)}$$

the spin density $S^\pm(\boldsymbol{r},t)$ is rewritten as

$$\begin{aligned}
S^\pm(\boldsymbol{r},t) &= \frac{8e^2\hbar^3}{m_{\rm e}^2}\mathscr{A}^{({\rm so})z}_\mu(\boldsymbol{r},t)\mathscr{A}^{({\rm ex})\pm}_\nu(\boldsymbol{r}){\rm Im}\sum_{\sigma=\pm}\sigma\sum_{\boldsymbol{k}}\sum_\omega f_\omega k_\mu k_\nu g^{\rm a}_{\boldsymbol{k},\omega,-\sigma}(g^{\rm a}_{\boldsymbol{k},\omega,\sigma})^2 \\
&+\frac{4e^2\hbar^3 J_{\rm ex}}{m_{\rm e}^2}\frac{\partial\mathscr{A}^{({\rm so})z}_\mu(\boldsymbol{r},t)}{\partial t}\mathscr{A}^{({\rm ex})\pm}_\nu(\boldsymbol{r})\sum_{\sigma=\pm}\sum_{\boldsymbol{k}}\sum_\omega k_\mu k_\nu\Bigg\{\frac{df_\omega}{d\omega}{\rm Re}(g^{\rm a}_{\boldsymbol{k},\omega,\sigma}-g^{\rm r}_{\boldsymbol{k},\omega,\sigma})(g^{\rm a}_{\boldsymbol{k},\omega,-\sigma})^2 g^{\rm a}_{\boldsymbol{k},\omega,\sigma} \\
&\pm i{\rm Im}\Big[\sigma\frac{df_\omega}{d\omega}g^{\rm r}_{\boldsymbol{k},\omega,\sigma}(g^{\rm a}_{\boldsymbol{k},\omega,-\sigma})^2 g^{\rm a}_{\boldsymbol{k},\omega,\sigma}+2\hbar J_{\rm ex}f_\omega(g^{\rm a}_{\boldsymbol{k},\omega,-\sigma})^3(g^{\rm a}_{\boldsymbol{k},\omega,\sigma})^3\Big]\Bigg\}.
\end{aligned} \qquad \text{(S.3)}$$

Here we neglect contributions from the products of $g^{\rm a}$ to calculate terms proportional to the first order in Ω as they only give small corrections compared with those from the products of $g^{\rm r}$ and $g^{\rm a}$. Therefore, the spin density reduces to

$$\begin{aligned}
S^\pm(\boldsymbol{r},t) &= \frac{8e^2\hbar^3}{m_{\rm e}^2}\mathscr{A}^{({\rm ex})\pm}_\nu(\boldsymbol{r})\Bigg[\mathscr{A}^{({\rm so})z}_\mu(\boldsymbol{r},t){\rm Im}\sum_{\sigma=\pm}\sigma\sum_{\boldsymbol{k}}\sum_\omega f_\omega k_\mu k_\nu g^{\rm a}_{\boldsymbol{k},\omega,-\sigma}(g^{\rm a}_{\boldsymbol{k},\omega,\sigma})^2 \\
&-\frac{J_{\rm ex}}{2}\frac{\partial\mathscr{A}^{({\rm so})z}_\mu(\boldsymbol{r},t)}{\partial t}\sum_{\sigma=\pm}({\rm Re}\mp i\sigma{\rm Im})\sum_{\boldsymbol{k}}\sum_\omega\frac{df_\omega}{d\omega}k_\mu k_\nu g^{\rm r}_{\boldsymbol{k},\omega,\sigma}(g^{\rm a}_{\boldsymbol{k},\omega,-\sigma})^2 g^{\rm a}_{\boldsymbol{k},\omega,\sigma}\Bigg] \\
&= \frac{8e^2\hbar}{m_{\rm e}}\mathscr{A}^{({\rm ex})\pm}_\nu(\boldsymbol{r})\Bigg\{C^{(1)}_{\mu\nu}\mathscr{A}^{({\rm so})z}_\mu(\boldsymbol{r},t)-\frac{J_{\rm ex}}{2}\Big[C^{(2)}_{\mu\nu}\mp iC^{(3)}_{\mu\nu}\Big]\frac{\partial\mathscr{A}^{({\rm so})z}_\mu(\boldsymbol{r},t)}{\partial t}\Bigg\}.
\end{aligned} \qquad \text{(S.4)}$$

The summations over $\boldsymbol{k}$ and ω are performed in the following manner,

$$\begin{aligned}
C^{(1)}_{\mu\nu} &= \frac{\hbar^2}{m_{\rm e}}{\rm Im}\sum_{\sigma=\pm}\sigma\sum_{\boldsymbol{k}}\sum_\omega f_\omega k_\mu k_\nu g^{\rm a}_{\boldsymbol{k},\omega,-\sigma}(g^{\rm a}_{\boldsymbol{k},\omega,\sigma})^2 \\
&= \frac{\nu_{\rm e}}{2\pi}\delta_{\mu\nu}{\rm Im}\sum_{\sigma=\pm}\int_0^\infty d\varepsilon\int_{-\infty}^0 d\omega\,\frac{\varepsilon}{(\hbar\omega-\varepsilon+\varepsilon_{{\rm F},-\sigma}-i\eta)(\hbar\omega-\varepsilon+\varepsilon_{{\rm F},\sigma}-i\eta)^2} \\
&= \frac{\nu_{\rm e}}{4\pi\hbar J_{\rm ex}^2}\delta_{\mu\nu}{\rm Im}\sum_{\sigma=\pm}\sigma\int_{-\infty}^0 d\varepsilon\int_{-\infty}^0 d\omega\,\frac{\varepsilon-\sigma J_{\rm ex}}{\omega+\varepsilon+\varepsilon_{{\rm F},\sigma}-i\eta} \\
&= \frac{\nu_{\rm e}}{4\pi\hbar J_{\rm ex}^2}\delta_{\mu\nu}{\rm Im}\sum_{\sigma=\pm}\sigma\int_{-\infty}^{-\sigma J_{\rm ex}} d\varepsilon\int_{-\infty}^0 d\omega\,\frac{\varepsilon}{\omega+\varepsilon+\varepsilon_{\rm F}-i\eta} \\
&= -\frac{\nu_{\rm e}}{4\pi\hbar J_{\rm ex}^2}\delta_{\mu\nu}{\rm Im}\int_{-J_{\rm ex}}^{J_{\rm ex}} d\varepsilon\int_{-\infty}^0 d\omega\,\frac{\varepsilon}{\omega+\varepsilon+\varepsilon_{\rm F}-i\eta} \\
&= -\frac{\nu_{\rm e}}{4\pi\hbar J_{\rm ex}^2}\delta_{\mu\nu}{\rm Im}\int_{-J_{\rm ex}}^{J_{\rm ex}} d\varepsilon\,\varepsilon\Big[\ln(\varepsilon+\varepsilon_{\rm F}-i\eta)+i\pi\Big] \\
&= -\frac{\nu_{\rm e}\eta}{16\pi\tau J_{\rm ex}}\delta_{\mu\nu}\Bigg\{\frac{\varepsilon_{\rm F}}{J_{\rm ex}}\ln\left[\frac{(\varepsilon_{\rm F}+J_{\rm ex})^2+\eta^2}{(\varepsilon_{\rm F}-J_{\rm ex})^2+\eta^2}\right]-2 \\
&+\frac{\varepsilon_{\rm F}^2-J_{\rm ex}^2-\eta^2}{J_{\rm ex}\eta}\left[\tan^{-1}\left(\frac{\eta}{\varepsilon_{\rm F}+J_{\rm ex}}\right)-\tan^{-1}\left(\frac{\eta}{\varepsilon_{\rm F}-J_{\rm ex}}\right)\right]\Bigg\},
\end{aligned} \qquad \text{(S.5)}$$

$$\begin{aligned}
C^{(2)}_{\mu\nu} &= \frac{\hbar^2}{m_{\rm e}}{\rm Re}\sum_{\sigma=\pm}\sum_{\boldsymbol{k}}\sum_\omega\frac{df_\omega}{d\omega}k_\mu k_\nu g^{\rm r}_{\boldsymbol{k},\omega,\sigma}(g^{\rm a}_{\boldsymbol{k},\omega,-\sigma})^2 g^{\rm a}_{\boldsymbol{k},\omega,\sigma} \\
&= \frac{\nu_{\rm e}}{2\pi}\delta_{\mu\nu}{\rm Re}\sum_{\sigma=\pm}\int_{-\infty}^0 d\varepsilon\,\frac{\varepsilon}{(\varepsilon+\varepsilon_{{\rm F},\sigma}+i\eta)(\varepsilon+\varepsilon_{{\rm F},-\sigma}-i\eta)^2(\varepsilon+\varepsilon_{{\rm F},\sigma}-i\eta)}
\end{aligned}$$

$$
\begin{aligned}
&= \frac{\nu_{\rm e}}{8\pi J_{\rm ex}}\delta_{\mu\nu}{\rm Re}\sum_{\sigma=\pm}\sigma\int_{-\infty}^{0}d\varepsilon\left[\frac{\sigma\varepsilon_{\rm F}J_{\rm ex}}{i2\eta(\sigma J_{\rm ex}-i\eta)^2}\left(\frac{1}{\varepsilon+\varepsilon_{\rm F}-\sigma J_{\rm ex}+i\eta}-\frac{1}{\varepsilon+\varepsilon_{\rm F}+\sigma J_{\rm ex}-i\eta}\right)\right.\\
&\quad\left.-\frac{1}{i2\eta}\left(\frac{1}{\varepsilon+\varepsilon_{\rm F}-\sigma J_{\rm ex}+i\eta}-\frac{1}{\varepsilon+\varepsilon_{\rm F}-\sigma J_{\rm ex}-i\eta}\right)-\frac{\varepsilon_{\rm F}-\sigma J_{\rm ex}+i\eta}{\sigma J_{\rm ex}-i\eta}\frac{1}{(\varepsilon+\varepsilon_{\rm F}-\sigma J_{\rm ex}+i\eta)^2}\right]\\
&= \frac{\nu_{\rm e}}{8\pi J_{\rm ex}}\delta_{\mu\nu}{\rm Re}\sum_{\sigma=\pm}\sigma\left\{\frac{\sigma\varepsilon_{\rm F}J_{\rm ex}}{i2\eta(\sigma J_{\rm ex}-i\eta)^2}\left[\ln\left(\frac{\varepsilon_{\rm F}-\sigma J_{\rm ex}+i\eta}{\varepsilon_{\rm F}+\sigma J_{\rm ex}-i\eta}\right)-i2\pi\right]\right.\\
&\quad\left.-\frac{1}{i2\eta}\left[\ln\left(\frac{\varepsilon_{\rm F}-\sigma J_{\rm ex}+i\eta}{\varepsilon_{\rm F}-\sigma J_{\rm ex}-i\eta}\right)-i2\pi\right]+\frac{1}{\sigma J_{\rm ex}-i\eta}\right\}\\
&= -\frac{\nu_{\rm e}\tau\varepsilon_{\rm F}(J_{\rm ex}^2-\eta^2)}{2\hbar(J_{\rm ex}^2+\eta^2)^2}\delta_{\mu\nu}\left\{1-\frac{\eta(J_{\rm ex}^2+\eta^2)}{\pi\varepsilon_{\rm F}(J_{\rm ex}^2-\eta^2)}+\frac{\eta J_{\rm ex}}{2\pi(J_{\rm ex}^2-\eta^2)}\ln\left[\frac{(\varepsilon_{\rm F}+J_{\rm ex})^2+\eta^2}{(\varepsilon_{\rm F}-J_{\rm ex})^2+\eta^2}\right]\right.\\
&\quad-\frac{1}{2\pi}\left[\tan^{-1}\left(\frac{\eta}{\varepsilon_{\rm F}+J_{\rm ex}}\right)+\tan^{-1}\left(\frac{\eta}{\varepsilon_{\rm F}-J_{\rm ex}}\right)\right]\\
&\quad\left.-\frac{(J_{\rm ex}^2+\eta^2)^2}{2\pi\varepsilon_{\rm F}J_{\rm ex}(J_{\rm ex}^2-\eta^2)}\left[\tan^{-1}\left(\frac{\eta}{\varepsilon_{\rm F}+J_{\rm ex}}\right)-\tan^{-1}\left(\frac{\eta}{\varepsilon_{\rm F}-J_{\rm ex}}\right)\right]\right\}, \qquad \text{(S.6)}
\end{aligned}
$$

$$
\begin{aligned}
C_{\mu\nu}^{(3)} &= \frac{\hbar^2}{m_{\rm e}}{\rm Im}\sum_{\sigma=\pm}\sigma\sum_{\boldsymbol{k}}\sum_{\omega}\frac{df_\omega}{d\omega}k_\mu k_\nu g^{\rm r}_{\boldsymbol{k},\omega,\sigma}(g^{\rm a}_{\boldsymbol{k},\omega,-\sigma})^2 g^{\rm a}_{\boldsymbol{k},\omega,\sigma}\\
&= \frac{\nu_{\rm e}}{8\pi J_{\rm ex}}\delta_{\mu\nu}{\rm Im}\sum_{\sigma=\pm}\int_{-\infty}^{0}d\varepsilon\left[\frac{\varepsilon_{\rm F}(\sigma 2J_{\rm ex}-i\eta)}{\sigma 2J_{\rm ex}(\sigma J_{\rm ex}-i\eta)^2}\left(\frac{1}{\varepsilon+\varepsilon_{\rm F}-\sigma J_{\rm ex}+i\eta}-\frac{1}{\varepsilon+\varepsilon_{\rm F}+\sigma J_{\rm ex}-i\eta}\right)\right.\\
&\quad\left.-\frac{1}{\sigma 2J_{\rm ex}}\left(\frac{1}{\varepsilon+\varepsilon_{\rm F}-\sigma J_{\rm ex}+i\eta}-\frac{1}{\varepsilon+\varepsilon_{\rm F}-\sigma J_{\rm ex}-i\eta}\right)-\frac{\varepsilon_{\rm F}-\sigma J_{\rm ex}+i\eta}{\sigma J_{\rm ex}-i\eta}\frac{1}{(\varepsilon+\varepsilon_{\rm F}-\sigma J_{\rm ex}+i\eta)^2}\right]\\
&= \frac{\nu_{\rm e}}{8\pi J_{\rm ex}}\delta_{\mu\nu}{\rm Im}\sum_{\sigma=\pm}\left\{\frac{\varepsilon_{\rm F}(\sigma 2J_{\rm ex}-i\eta)}{\sigma 2J_{\rm ex}(\sigma J_{\rm ex}-i\eta)^2}\left[\ln\left(\frac{\varepsilon_{\rm F}-\sigma J_{\rm ex}+i\eta}{\varepsilon_{\rm F}+\sigma J_{\rm ex}-i\eta}\right)-i2\pi\right]\right.\\
&\quad\left.-\frac{1}{\sigma 2J_{\rm ex}}\left[\ln\left(\frac{\varepsilon_{\rm F}-\sigma J_{\rm ex}+i\eta}{\varepsilon_{\rm F}-\sigma J_{\rm ex}-i\eta}\right)-i2\pi\right]+\frac{1}{\sigma J_{\rm ex}-i\eta}\right\}\\
&= -\frac{\nu_{\rm e}\varepsilon_{\rm F}J_{\rm ex}}{2(J_{\rm ex}^2+\eta^2)^2}\delta_{\mu\nu}\left\{1-\frac{\eta(J_{\rm ex}^2+\eta^2)}{2\pi\varepsilon_{\rm F}J_{\rm ex}^2}+\frac{\eta(3J_{\rm ex}^2+\eta^2)}{8\pi J_{\rm ex}^3}\ln\left[\frac{(\varepsilon_{\rm F}+J_{\rm ex})^2+\eta^2}{(\varepsilon_{\rm F}-J_{\rm ex})^2+\eta^2}\right]\right.\\
&\quad-\frac{1}{2\pi}\left[\tan^{-1}\left(\frac{\eta}{\varepsilon_{\rm F}+J_{\rm ex}}\right)+\tan^{-1}\left(\frac{\eta}{\varepsilon_{\rm F}-J_{\rm ex}}\right)\right]\\
&\quad\left.-\frac{(J_{\rm ex}^2+\eta^2)^2}{4\pi\varepsilon_{\rm F}J_{\rm ex}^3}\left[\tan^{-1}\left(\frac{\eta}{\varepsilon_{\rm F}+J_{\rm ex}}\right)-\tan^{-1}\left(\frac{\eta}{\varepsilon_{\rm F}-J_{\rm ex}}\right)\right]\right\}. \qquad \text{(S.7)}
\end{aligned}
$$
